# Efficacy Prediction of Four Pharmaceutical Formulations for Intramammary Administration Containing *Aloe vera* (L.) Burm. f. Combined With Ceftiofur or Cloxacillin in Lactating Cows as an Alternative Therapy to Treat Mastitis Caused by *Staphylococcus aureus*

**DOI:** 10.3389/fvets.2021.572568

**Published:** 2021-03-22

**Authors:** Natalia Forno-Bell, Marcos A. Munoz, Oscar Chacón, Paulina Pachá, Daniela Iragüen, Javiera Cornejo, Betty San Martín

**Affiliations:** ^1^Laboratorio de Farmacología Veterinaria, Facultad de Ciencias Veterinarias y Pecuarias, Universidad de Chile, Santiago, Chile; ^2^Programa de Doctorado en Ciencias Silvoagropecuarias y Veterinarias, Campus Sur, Universidad de Chile, Santiago, Chile; ^3^Laboratorio de Investigación en Mastitis y Calidad de Leche, Departamento de Ciencia Animal, Facultad de Ciencias Veterinarias, Universidad de Concepción, Chillán, Chile; ^4^Escuela de Medicina Veterinaria, Facultad de Recursos Naturales y Medicina Veterinaria, Universidad Santo Tomás, Temuco, Chile; ^5^Departamento de Ciencias Clínicas, Facultad de Ciencias Veterinarias y Pecuarias, Universidad de Chile, Santiago, Chile

**Keywords:** cow mastitis, *Staphyhcoccus aureus*, *Aloe vera (L.)* Burm. f., cloxacillin, ceftiofur, intramammary formulations, efficacy index

## Abstract

Synergy or additive effect between *Aloe vera* (L.) Burm.f. and beta-lactam (β-lactam) antibiotics has been reported against *Staphylococcus aureus*, one of the most important etiological agents of cow mastitis. The goal of the present study was to predict the efficacy of intramammary formulations containing the *Aloe vera* gel extract in the combination with cloxacillin or ceftiofur at low concentrations in lactating cows as an alternative therapy. Each quarter of 20 healthy Holstein Friesian lactating cows were treated with a single dose of one of the following formulations, corresponding to one of these treatment groups: A1, A2, A3, and A4. A1 and A2 contained cloxacillin at 0.25 and 0.5 mg/ml, whereas A3 and A4 contained ceftiofur 0.25 and 0.5 mg/ml, respectively. In addition, all formulations contained 600 mg/ml of an alcoholic extract of *Aloe vera*. Milk samples were taken at predefined time points. Antibiotics and aloin (active compound of *Aloe vera*) concentrations were assessed by liquid chromatography mass spectrometry system (LC-MS/MS). Pharmacokinetic profiles were obtained, and the efficacy index, the fraction of dosing interval in which the antimicrobial concentration remains above the minimum inhibitory concentrations (MICs) (T > MIC) for each formulation, was calculated considering MIC values against *Staphylococcus aureus* ATCC 29213 as obtained for the combination *Aloe vera* + antibiotic and aloin concentration in the extract. Mammary gland safety assessment was performed for each combination. Values of the main efficacy index for this study, T > MIC (h) for *Aloe vera* were 23.29, 10.50, 27.50, and 13.89, whereas for cloxacillin or ceftiofur were 19.20, 10.9, 19.74, and 15.63, for A1, A2, A3, and A4, respectively. Only A1 and A3 reached aloin and antibiotic recommended values as predictors of clinical efficacy for cloxacillin, ceftiofur, and aloin (50, 70, and 60%, respectively), assuming a dose interval of 24 h. The efficacy index values obtained suggest that A1 and A3 might be an effective therapy to treat bovine mastitis caused by *S. aureus* after a single dose. Nevertheless, further trials in *S. aureus* mastitis clinical cases are mandatory to confirm the efficacy of *Aloe vera* formulations.

## Introduction

*Aloe vera (L.)* Burm. f is a popular plant species within the Asphodelaceae Juss. family (1). It is indigenous to Africa and the Arabian Peninsula, where they have been traditionally used to treat bacterial and parasitic infections in both domestic animals and humans (2). Over the years, such antibacterial activity has been assessed by several researcher groups. For instance, some studies have proved the *in vitro* effectiveness of *Aloe vera* gel derivates against *Escherichia coli, Klebsiella pneumoniae*, and *Staphylococcus aureus* bacteria, which are the most commonly found agents in cases of mastitis of dairy cows (3–9).

These results are interesting when considering that the main reasons for using antimicrobials in dairy cattle are the treatment of clinical cases of mastitis and the implementation of dry-cow prophylactic therapies (4, 10, 11). However, when treating mastitis cases caused by infections with *S. aureus* strains, cure rates are lower than when other bacterial species are involved (12). This treatment failure may be explained by several virulence factors, such as the type of bacterial toxins released, degree of tissue invasion, immune suppression, resistance to neutrophils, the ability of forming biofilms that protect bacteria from the effects of antimicrobials, and bacterial resistance to antimicrobials, especially beta-lactams (β-lactams) (13, 14). Naturally, the poor efficacy of current therapies against mastitis caused by *S. aureus* and the worldwide concern about the rise of antimicrobial resistance are likely the major forces behind the growing interest in developing alternative treatment to antibiotics for mastitis cases.

*Aloe vera* is one of the new therapeutic alternatives proposed as an antibacterial agent (15, 16). *Aloe vera* is a xerophyte succulent plant known for its medicinal properties. These properties are especially attributed to its inner gel (17), which is a transparent and mucilaginous jelly-like substance comprised of water (99% w/w), polysaccharides, sugars, aminoacids, enzymes, minerals, vitamins, and phenolic compounds (18). Among these components, polysaccharides are the main fraction in terms of dry matter and important reported therapeutic properties, such as antitumoral, anti-inflammatory, and immunosuppressive activities (17). Regarding antibacterial activity of *Aloe vera* gel, it has been mainly attributed to phenolic compounds, such as anthraquinones (19–21). However, some studies have also proposed that such antibacterial activity may result from a synergistic interaction of several of its compounds (6, 17, 18).

In the case of anthraquinones, some *in vitro* studies showed that these compounds cause membrane cell damages on methicillin-resistant *S. aureus* (MRSA) that are unrelated to the expression of certain genes responsible for cell wall synthesis. Instead, the damage would be a result of a strong specific affinity between anthraquinones (i.e., emodin and aloin) and the phospholipids present in bacterial membranes—such as phosphatidylglycerol—and it would trigger an increase in membrane permeability (22–24). In an attempt to take advantage of its medicinal properties, a commercial intramammary formulation of *Aloe vera* gel mixed with diverse other plant extracts and essential oils (Mastiliber^®^) has been developed in Mexico. Unfortunately, even though this formulation can result in a clinical cure of mastitis cases due to the anti-inflammatory properties of these plant extracts and oils, some researchers have found that this product could not achieve a bacteriological cure because it did not completely inhibit the bacterial growth of isolates sourced from clinically affected cows (25).

However, some studies show evidence of the synergistic interactions between medicinal plants and common antimicrobials when used against *S. aureus* (26–28). In the case of *Aloe vera*, Chacón et al. (29) found that the extract from this plant presented a synergistic or additive effect when combined with some β-lactam drugs, such as cloxacillin and ceftiofur, respectively. These interactions observed between the *Aloe vera* extract and the antimicrobial drugs result in lower minimum inhibitory concentrations (MICs) for the combined formulations than for each antimicrobial on its own when used against *S. aureus*. This is interesting because these drugs are commonly used to treat clinical mastitis caused by *S. aureus* (30, 31). Therefore, this evidence suggests that formulations including *Aloe vera* extracts along with low concentrations of ceftiofur or cloxacillin could be suitable for the treatment of clinical mastitis *via* intramammary syringes. Nevertheless, to adequately predict the efficacy of any antimicrobial drug, its pharmacokinetic and pharmacodynamic (PK/PD) properties must be evaluated. To this end, different PK/PD indices are used to associate the susceptibility of an infectious agent with the level of exposure of that agent to an antimicrobial drug. Such association is quantified on the basis of the MIC observed for those bacteria. In particular, the three most commonly used indices are: the ratio between the maximum concentration reached of an antimicrobial and the MIC value (*C*_max_/MIC), the fraction of dosing interval in which the antimicrobial concentration remains above the MIC (T > MIC), and the ratio between the area under the concentration–time curve and the MIC value (AUC/MIC). The process of calculating these efficacy indices has become the gold standard for both the assessment of PK/PD and as a guide for the establishment of dosing regimens (32–34).

In light of the information above, this study is aimed to predict the efficacy of four formulations combining the *Aloe vera* gel extract and low concentrations of ceftiofur or cloxacillin, as an intramammary therapeutic alternative for the treatment of clinical mastitis caused by *S. aureus* in lactating dairy cows.

## Materials and Methods

The experimental stage of this work was located on a dairy farm in the area of the city of Chillán, Chile, where all animals were subjected to procedures and management protocols that followed the guidelines established in accordance with—and approved by—the Institutional Committee for Animal Care and Use (CICUA, by its Spanish acronym) from the University de Chile (certificate number: 17004 VET-UCH).

Milk sample cultures and somatic cell count (SCC) evaluation were done following the National Mastitis Council standards (35) in the Mastitis and Milk Quality Research Laboratory (MMQRL) of the Facultad de Ciencias Veterinarias, Universidad de Concepción, Chile.

As for the pharmacological laboratory procedures, these were performed at the Laboratory of Veterinary Pharmacology, which is part of the Facultad de Ciencias Veterinarias y Pecuarias from the Universidad de Chile. This laboratory has been accredited under the ISO 17025 norm (36).

### Animals

The animals used for this experiment were 20 lactating cows of Holstein Friesian and Normande breeds. All these cows were intramammary clinically healthy from both clinical and subclinical evaluation. According to guidelines from the European Medicines Agency (EMEA) (37), cows were enrolled only if all the quarters SCC values were <200,000 cells/ml and their milk tested no growth for pathogenic or contaminant microorganisms. Those cows found eligible for this study received no other drug treatment for at least 20 days before entering the experiment. In addition, the health status of the mammary glands of these cow was on-farm reassessed before performing the safety test by using a DeLaval DCC^®^ device (DeLaval DCC, Tumba, Sweden), and only cows whose quarters-composite SCC value was <100,000 cells/ml were included in the experiment.

As a first approach for the assessment of effectiveness on the use of these combinations, to perform PK and safety studies, no cows were inoculated with *S. aureus*. Furthermore, every quarter of each cow was considered as an independent unit following the split-udder model (38–40) to accomplish the animal welfare reduction criteria of both CICUA and European Communities Council Directive protocols.

### Treatments and Sampling Procedures

Four treatment formulations were prepared and assigned to four corresponding treatment groups A1, A2, A3, and A4. All these formulations contained 600 mg/ml of *Aloe vera* gel extract and differed only by the antimicrobial drug and concentration used. Formulations for groups A1 and A2 contained 0.25 and 0.5 mg/ml of cloxacillin, respectively, whereas formulations for groups A3 and A4 contained 0.25 and 0.5 mg/ml of ceftiofur, respectively. Each cow enrolled in the study was then assigned to one of these four treatment groups (namely: A1, A2, A3, and A4). Every treatment group was assigned to a single cow. Every quarter of all the cows received exclusively a single intramammary dose of the antimicrobial treatment previously allocated to their group. The treatments were applied after milking of the cows following the recommendations of the National Mastitis Council (41). Briefly, the treatment began with a rigorous disinfection of each teat and teat-end and performing only a partial insertion of the treatment syringe into the streak canal.

Milk samples were collected from each quarter at six sampling times (0, 3, 6, 9, 12, and 24 h) and then stored at −20°C while waiting for further processing by the liquid chromatography mass spectrometry system (LC-MS/MS) chromatography.

No inclusion control groups were considered in the experimental design because this study only intended to predict the efficacy of the intramammary formulations containing cloxacillin or ceftiofur, but at lower concentrations than current commercially available products; as these antimicrobials show a synergistic or additive effect with the *Aloe vera* gel extract that was part of the formulations.

### Safety Assessment

For the safety assessment, two treated cows were randomly selected from each group, of the four treatment groups, and then monitored every 24 h for 4 days after treatment administration using the EMEA standards (37). The milk samples were obtained aseptically from each quarter of these two cows and then observed looking for changes in appearance that would be consistent with clinical mastitis, such as an altered milk color or the presence of flecks and clots. In addition, these samples were plated in duplicate onto 5% sheep-blood agar with aesculin and then checked for bacterial growth after an incubation time of 24 and 48 h at 37°C. Whenever more than three types of microorganisms grew in a plate, that sample was considered contaminated (41).

All milk samples destined for SCC were collected with bronopol and stored overnight at 4°C waiting for further analysis by the flow cytometry technique. These analyses were performed at the Central Laboratory of the Animal Nutrition and Environment from the Institute of Agricultural Research of Chile (INIA, by its Spanish acronym) in the city of Temuco, Chile.

### Intramammary Formulations

The procedure of methanol extraction on the freeze-dried powder of *Aloe vera* gel consisted of measuring 5 g of powder that was placed in a plastic tube and then diluted in a solution of 40-ml of methanol and 0.5 ml of acetic acid. This sample was vortexed, sonicated, and then centrifuged at 492 × *g* for 10 min. The resulting supernatant was then collected in a bottom flask, while the remaining sediment was used to repeat the extraction process. The sum of supernatants collected from both extractions was concentrated using a rotavapor Hei Vap Precision ML/G3, (Heidolph^®^ Schwabach, Germany), following the procedure described by Habeeb et al. (3) with modifications. Lastly, the pH of the extract was adjusted to 5.0 ± 0.3 by using sodium hydroxide and then stored at 4°C. The final concentration of the *Aloe vera* extract produced was of 1,200 mg/ml.

As for the methanol residues, these were detected by using the headspace gas chromatography with flame ionization (HS-GC-FID) technique. This analysis was performed at the Studies Center for the Development of Chemistry (CEPEDEQ, by its Spanish acronym), dependent on the Faculty of Chemistry and Pharmaceutics from the Universidad de Chile.

Finally, four intramammary formulations were prepared by aqueous dilution of the extract (600 mg/ml), and either cloxacillin or ceftiofur (at concentrations of 0.25 or 0.5 mg/ml) to complete 10-ml dose syringes, of the four respective treatments.

### Chemical Reagents and Standards

The *Aloe vera* freeze-dried gel powder used to prepare the intramammary formulations was purchased from America Inc. (District Court, Scottsdale, AZ, USA). The cloxacillin and ceftiofur standards were of 99% purity and sourced from Dr. Ehrenstorfer^®^ (Augsburg, Germany). As for aloin A and aloin B standards, these were of 87 and 92.9% purity, respectively and were manufactured by ChromaDex Inc. (Irvine, CA, USA).

Other reagents, such as high performance liquid chromatography (HPLC)-grade methanol, analytical-grade acetic acid, analytical-grade ethyl acetate, analytical-grade ethanol, analytical-grade acetonitrile, analytical-grade hexane, analytical-grade acetic acid, analytical-grade sodium chloride, and analytical-grade sodium sulfate were sourced from Merck (Darmstadt, Germany).

### Analytical Procedure

#### Standards and Working Solutions

Standard aloin A and aloin B solutions were prepared by dissolving these drugs in a solution comprised of HPLC-grade methanol/water and methanol (90:10, v/v). As for the cloxacillin and ceftiofur standard solutions, these were prepared by a solution of HPLC-grade water/acetonitrile (50:50, v/v). Once prepared, these solutions (1 mg/ml) were stored in the dark and refrigerated at a temperature of 4 ± 2°C for <3 months.

#### Sample Preparation

##### Concentrations of Aloin A and Aloin B in *Aloe vera* Gel Extract

Aloin A and aloin B were quantified from *Aloe vera* gel extract using a procedure based on the one described by Elsohly et al. (42). Briefly, 0.5 g of the extract was weighed in within a 50-ml falcon tube, in which we then poured a mixture of 1 ml of ethanol, 2 ml of saturated saline solution, and 4 ml of an ethyl-acetate/methanol solution (90:10, v/v). The samples were vortexed, sonicated, and centrifuged at 492 × *g* for 5 min. Then, the supernatant was collected in a clean tube, meanwhile the remaining sediment was subjected to the same procedure twice, by adding 2 ml of the ethyl-acetate/methanol solution, and then all resulting supernatants were combined in the same tube. Afterward, samples were dried out under a stream of nitrogen at 27 ± 5°C and reconstituted with 0.5 ml of methanol. Then, the samples were vortexed and sonicated for 5 min, and the resulting solutions were transferred to the Eppendorf Tubes (Hamburg, Germany). Lastly, these tubes were centrifuged at 11,337 × *g* for 5 min, and the resulting supernatants were transferred to LC vials. From these vials, a volume of 20 μl was injected into the LC-MS/MS to set apart and identify the analytes.

For this method, the limit of detection (LOD) (S/N = 3:1) for both aloin A and aloin B was established as 3 ng/ml, whereas the limits of quantification (LOQ) (S/N = 10:1) were established as 10 and 15 ng/ml for aloin A and B, respectively.

##### Concentrations of Aloin A, Aloin B, Cloxacillin, and Ceftiofur in Milk

Aloin A, aloin B, cloxacillin, and ceftiofur were extracted from the milk using a procedure based on the one described by Karami-Osboo et al. (43). First, 5 g of milk were weighed in within a 50-ml falcon tube, in which we then poured a mixture of 15 ml of acetonitrile, 3.5 g of sodium chloride, and 1 g of sodium sulfate. The samples were vortexed, sonicated, and centrifuged at 492 × *g* for 5 min. Then, the supernatant was collected in a clean tube where it was mixed with 20 ml of hexane, and then the solutions were agitated and centrifuged at 492 × *g* for 5 min. The resulting supernatants were then discarded, dried out under nitrogen at 30 ± 2°C, and then reconstituted with 0.5 ml of methanol/water solution (60:40, v/v). The tubes were again vortexed and sonicated for 5 min, and their contents were transferred to 1.5-m Eppendorf tubes. These tubes were centrifuged at 13,200 × *g* for 5 min, and the resulting supernatants were transferred to LC vials. From these vials, aliquots of 20 μl were injected into the LC-MS/MS.

For this method, the LOD for both aloins (A and B), cloxacillin, and ceftiofur were established at 0.5 ng/ml, 0.05, and 0.01 μg/mL, respectively. In the case of the LOQ, these were established at 1 ng/ml, 0.1, and 0.05 μg/ml for aloin A, aloin B, cloxacillin, and ceftiofur, respectively.

#### Instrumental Analysis

##### Liquid Chromatography

Samples were analyzed using an LC Agilent 1200 System, equipped with a deaerator, an automatic sample injector, and a binary pump (Agilent, Waldbronn, Germany). A Sunfire C18 column of 2.1 mm × 3.5 μm × 150 mm (Waters^®^, Milford, MA, USA) was used for the chromatographic separation of the analytes. The mobile phase comprised two solutions: one of water/acetic acid (90:10, v/v) and another of water/acetonitrile/acetic acid (10:80:10, v/v). The chromatographic analysis was run at 35°C using a flow rate of 0.2 ml/min. Under these chromatographic conditions, the retention times were established at 8.88, 8.33, 12.22, and 6.13 min for aloin A, aloin B, cloxacillin, and ceftiofur, respectively.

##### Mass Spectrometer

Analytes were identified using a Sciex API 5500 Mass Spectrometer (AB Sciex, Concord, ON, Canada). Aloin A and aloin B were isolated from the extract by ionization using a Turbo Spray ion-positive and ion-negative mode. The source temperature was 400°C, and the ion spray voltage was −4,200 and 4,200 V. The pressure was 15 psi for nitrogen gas curtain, 50 and 40 psi for ion source gases 1 and 2, respectively, and 4 psi for the collision gas.

As for the isolation of aloin A, aloin B, cloxacillin, and ceftiofur from milk by ionization, the modified conditions of the mass spectrometer were: source temperature at 600°C, ion spray at −4,500 and 5,500 V, and ion source gases 1 and 2 at 70 and 50 psi, respectively. Table 2 lists the product and precursor ions, declustering potential, entrance potential, collision energy, and cell exit potential.

The MS data were obtained in the multiple reaction monitoring (MRM) mode. Tables 1, 2 list the values for aloin A and aloin B detected in the gel extract, and the values for aloin A, aloin B, cloxacillin, and ceftiofur were detected in milk.

**Table 1:** Multiple reactions monitoring (MRM) for aloin A and aloin B detected in the extract.

	**Precursor ions**	**Product ions**	**DP**	**EP**	**CE**	**CXP**
Aloin A	419	239^†^	50	10	19	4
		211^‡^			19	4
Aloin B	419	239^†^	50	4	19	4
		211^‡^			19	3

*DP, declustering potential; EP, entrance potential; CE, collision energy; CXP, cell exit potential*.

† *Quantification ion*,

‡ *Confirmation ion*.

**Table 2:** Multiple reactions monitoring (MRM) for aloin A, aloin B, cloxacillin, and ceftiofur detected in milk.

	**Precursor ions**	**Product ions**	**DP**	**EP**	**CE**	**CXP**
Aloin A	419	239^†^	50	10	19	4
		211^‡^			19	4
Aloin B	419	239^†^	50	4	19	4
		211^‡^			19	3
Cloxacillin	436	160^†^	56	5	19	4
		277^‡^			19	4
Ceftiofur	524	285^†^	60	5	19	4
		241^‡^			19	4

*DP, declustering potential; EP, entrance potential, CE, collision energy, CXP, cell exit potential*.

† *Quantification ion*,

‡ *Confirmation ion*.

#### Validation of the Analytical Method

##### Extract Concentrations of Aloin A and Aloin B

Following the recommendations in the guidelines of the European Commission Decision 2002/657/EC (44), the analytical procedures were validated by LC-MS/MS before using them to determine the concentrations of aloin A and aloin B in the extract, as well as concentrations of aloin A, aloin B, cloxacillin, and ceftiofur in milk. To this end, the essential parameter values that were estimated to validate every analyte in both matrices were the specificity, recovery, repeatability, and intralaboratory reproducibility (Table 3).

**Table 3:** Validation parameters for aloin A and aloin B on *Aloe vera* extract samples and for aloin A, aloin B, cloxacillin, and ceftiofur on milk samples.

**Parameters**	**Values**					
	**Extract**			**Milk**		
	**AA**	**AB**	**AA**	**AB**	**CLX**	**CFT**
	**ng/ml**	**ng/ml**	**ng/ml**	**ng/ml**	**μg/ml**	**μg/ml**
Recovery (%)	87	85	87	86	90	85
Repeatability (CV%)	17	16	16	17	14	15
Intralaboratory reproducibility (CV%)	19	21	21	18	20	22
Calibration curves	20–320	20–320	1–20	1–20	0.1–4	0.05–2
			20–320	20–320		

*AA, aloin A; AB, aloin B; CLX, cloxacillin; CFT, ceftiofur*.

##### Quantification of Experimental Samples

The concentration of aloin A and aloin B in the extract and the concentration of aloin A, aloin B, cloxacillin, and ceftiofur in the milk were calculated using a linear regression equation. This equation was calculated based on matrix-matched calibration curves constructed using the standard solutions adjusted to different concentrations (*r* > 0.99). The concentration ranges that were determined for aloin A and aloin B in the extract were of 20–320 ng/ml, whereas the concentration ranges in milk for aloin A, aloin B, cloxacillin, and ceftiofur were of 1–20 ng/ml, 20–320 ng/ml, 0.1–4 μg/ml, and 0.05–2 μg/ml, respectively.

### Pharmacokinetics Analysis

The computer software PK Solutions 2.0 (Ashland, OH, USA) was used to fit the curve of concentrations in milk of aloin (A and B), cloxacillin, and ceftiofur vs. time. In addition, the PK parameters were determined using a non-compartmental analysis, through removing some of the assumptions related to a compartmental modeling but keeping the assumptions of instantaneous mixing and a constant volume for the sampling space (45).

The maximum concentration (*C*_max_) and time to *C*_max_ (*T*_max_) were determined on the basis of the curve plotted for the milk concentration over time in each quarter. In addition, the AUC for concentration vs. time was calculated using the trapezoidal method (46) from time zero to time of last measurable concentration. Finally, the elimination rate constant (λz) was calculated by the linear regression from the linear part to the terminal phase, and the terminal half-life (t1/2λz) was estimated as ln 2/λz.

### Minimum Inhibitory Concentration

A macrodilution test was performed to determine the MIC for the different combinations, following the recommendations from the CLSI (47).

*Aloe vera* gel extract (1,200 mg/ml) was diluted in Muller-Hinton II (Cation Adjusted) Broth (MHBCA) from BD^®^ (Franklin Lakes, NJ, USA) up to a range of concentrations from 150 to 1,200 mg/ml. In addition, the standard solutions of cloxacillin and ceftiofur were diluted in MHBCA to prepare 2-fold serial dilutions at a concentrations range of 0.015–128 μg/ml.

*Staphylococcus aureus* ATCC 29213 (KWIK-STIK) was sourced from Microbiologics^®^ (St. Cloud, MN, USA) and used to test the MIC for the different combinations of *Aloe vera* and cloxacillin or ceftiofur. First, *S. aureus* was rehydrated with Trypticase^™^ Soy Broth from BD^®^ (Franklin Lakes, NJ, USA). Then, these bacteria were cultured in the Trypticase^™^ Soy Broth from BD^®^ (Franklin Lakes, NJ, USA) with 5% sheep defibrinated blood (Franklin Lakes, NJ, USA) at an incubation temperature of 36 ± 1°C for 24 h. Once incubation ended, bacteria were preserved at −80°C using cryobank vials, while waiting for further processing. Later on and before proceeding to determine the MIC for each formulation, part of this *S. aureus* broth was thawed and grown overnight in a Plate Count Agar from BD^®^ (Franklin Lakes, NJ, USA) at 36°C. These cultured bacteria were then suspended in MHBCA from BD^®^ (Franklin Lakes, NJ, USA), and the optical density was adjusted according to the McFarland Standard Solution to standardize the number of bacteria. From this broth, dilutions containing 5 × 10^5^ UFC/ml were incubated along with dilutions of a combination of *Aloe vera* extract and antimicrobials. The MIC for the extract was set at the lowest concentration of the combination of the extract, cloxacillin, or ceftiofur that retained its inhibitory effect, thus resulting in no growth (i.e., broth shows no turbidity) of *S. aureus*. Additionally, *S. aureus* bacteria were incubated separately, in different tubes, with the extract, cloxacillin, and ceftiofur dilutions.

### Efficacy Prediction Indices

As no therapeutic efficacy indices have been recommended for aloin, the most frequently used indices were determined in this work for both aloin A and aloin B in each combination: T > MIC, *C*_max_/MIC, and AUC/MIC. In addition, the T > MIC index was calculated for cloxacillin and ceftiofur.

The *S. aureus* ATCC 29213 strain was challenged with the different combinations of the *Aloe vera* extract, cloxacillin, and ceftiofur for the purpose of calculating the MIC, and the calculation of the efficacy indices considered the concentration of aloin A and aloin B in the *Aloe vera* extract. It is worth noting that the recommended T > MIC values that have been linked to a good clinical efficacy for cloxacillin and ceftiofur are 50 and 70% of the dosing interval, respectively, whereas in the case of aloin, a 60% of this interval was considered (33).

### Statistical Analysis

Somatic cell score was calculated by performing a base-2 logarithmic (log2) transformation of the SCC values (48). Then, SCS values were subjected to a normality test (i.e., a Kolmogorov–Smirnov test), and the differences between treatment groups were assessed at different sampling times using a two-way repeated measures ANOVA. For statistical significance *p*-value was set as <0.05.

Data were analyzed using the SPSS version 23.0 statistical software (IBM Corp., Armonk, NY, USA).

## Results

### Characterization of *Aloe vera* Extract

The concentrations of aloin A and aloin B in the *Aloe vera* extract were 146.72 and 122.08 ng/ml, respectively.

Methanol concentration in the extract was 54 g/kg.

### Pharmacokinetics

The method described above was used to analyze milk samples collected from 20 quarters for each formulation, except for the treatment group A4 due to one dysfunctional teat.

**Figure 1:**
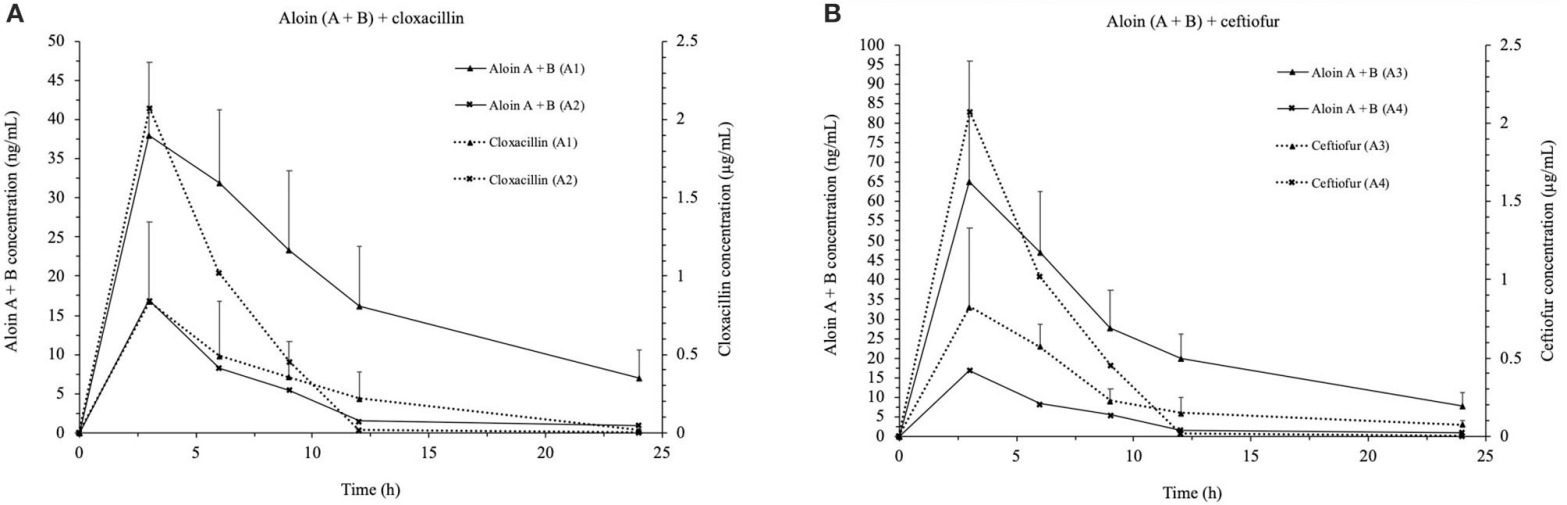
Comparative concentrations (mean ± SD, *n* = 20 for groups in A1, A2, and A3 and *n* = 19 for group A4) of aloin A + B and cloxacillin or ceftiofur in milk after a single dose administration of: A1, 600 mg/ml of *Aloe vera* + 0.25 mg/ml of cloxacillin **(A)**; A2, 600 mg/ml of *Aloe vera* + 0.5 mg/ml of cloxacillin **(A)**; A3, 600 mg/ml of *Aloe vera* + 0.25 mg/ml of ceftiofur **(B)**, and A4, 600 mg/ml of *Aloe vera* + 0.5 mg/ml of ceftiofur **(B)**, respectively.

Figure 1 shows the curve of concentrations of aloin (A + B) and cloxacillin in milk vs. time, after administering treatments A1 and A2, and also the curve of concentrations of aloin (A + B) and ceftiofur in milk vs. time, after administering treatments A3 and A4.

In all four groups, the higher concentration in milk of aloin (A + B), and cloxacillin or ceftiofur, peaked 3 h after the intramammary treatments were administered (*T*_max_). However, the four formulations contained the same concentration of the *Aloe vera* extract (600 mg/ml)—hence, they also contained the same concentration of aloin A and aloin B (109.07 ng/ml)—the maximum concentration (*C*_max_) of aloin (A + B) was different between treatments. In particular, the treatment group A3 showed the highest *C*_max_ for aloin (90.17 ± 54.98 ng/ml), whereas the treatment group A2 showed the lowest one (15.08 ± 10.85 ng/ml). Regarding the antimicrobials, the treatment group A2 showed the highest *C*_max_ for cloxacillin (4.17 ± 0.97 μg/ml), whereas the treatment group A4 showed the highest *C*_max_ for ceftiofur (1.02 ± 0.25 μg/ml). In contrast, the lowest *C*_max_ for cloxacillin and ceftiofur were observed in the treatment groups A1 (0.84 ± 0.51 μg/ml) and A3 (0.94 ± 0.34 μg/ml). Tables 4, 5 list the mean values for the PK parameters of each formulation.

**Table 4:** Mean ± SD (*n* = 20 per groups A1, A2, and A3 and *n* = 19 per group A4) for the pharmacokinetic parameters and therapeutic efficacy indices of aloin A + B (AB) detected in cow milk for each intramammary formulation of *Aloe vera* combined with cloxacillin or ceftiofur, after a single dose.

	**Aloin A** **+** **B**			
	**A1**	**A2**	**A3**	**A4**
*C*_max_	37.84 ± 12.31	15.08 ± 10.85	90.17 ± 54.98	59.08 ± 29.22
*T*_max_	3.45 ± 1.47	2.40 ± 1.23	3 ± 0	3 ± 0
AUC_last_	441.97 ± 45.93	107.54 ± 23.08	747.02 ± 97.74	337.26 ± 69.98
T1/2_el_	9.96 ± 4.12	9.10 ± 4.45	7.83 ± 2.55	2.94 ± 1.25
*C*_max_/MIC	10.34 ± 3.36	4.12 ± 2.96	24.64 ± 5.02	16.14 ± 3.98
AUC/MIC	120.76 ± 39.87	29.38 ± 7.37	204.10 ± 80.67	92.15 ± 23.71
T > MIC	23.29 ± 6.89	10.5 ± 4.82	27.5 ± 2.57	13.89 ± 3.90

*Peak milk concentration (C_max_, ng/ml), time to peak milk concentration (T_max_, h), curve of area under the concentration vs. time extrapolated to infinity (AUC_last_, ng-h/ml), elimination half-life (T_½_ el, h), minimum inhibitory concentration (MIC, ng/mL), relationship between the maximum concentration and MIC (C_max_/MIC), relation between the area under the curve and MIC (AUC/MIC), amount of time that concentration level is above MIC (T > MIC, h)*.

*Treatment groups correspond with the following formulations: A1 (600 mg/ml of Aloe vera + 0.25 mg/ml of cloxacillin), A2 (600 mg/ml of Aloe vera + 0.5 mg/ml of cloxacillin), A3 (600 mg/ml of Aloe vera + 0.25 mg/ml of ceftiofur), and A4 (600 mg/ml of Aloe vera + 0.5 mg/ml of ceftiofur)*.

*Efficacy indices of aloin A + B were calculated based on the minimum inhibitory concentration (MIC) of Aloe vera against S. aureus ATCC 29213 as calculated for the combination (15 mg/ml for Aloe vera) and the concentration of aloin A + aloin B in the Aloe vera extract (aloin MIC: 3.66 ng/ml)*.

**Table 5:** Mean ± SD (*n* = 20 per groups A1, A2, and A3 and *n* = 19 per group A4) for the pharmacokinetic parameters and therapeutic efficacy indices for cloxacillin (CLX) and ceftiofur (CFT) detected in cow milk for each formulation containing *Aloe vera* combined with cloxacillin or ceftiofur, after a single dose.

	**Cloxacillin**		**Ceftiofur**	
	**A1**	**A2**	**A3**	**A4**
*C*_max_	0.84 ± 0.51	4.17 ± 0.97	0.94 ± 0.34	1.02 ± 0.25
*T*_max_	3 ± 0	3.15 ± 0.67	3.6 ± 1.23	3.16 ± 0.69
AUC_last_	6.8 ± 2.31	10.78 ± 4.62	6.43 ± 1.56	5.76 ± 0.99
T1/2_el_	3.44 ± 0.58	1.65 ± 0.92	9.56 ± 6.18	5.74 ± 1.82
T > MIC	19.20 ± 3.12	10.90 ± 0.31	19.74 ± 6.36	15.63 ± 3.71

*Peak milk concentration (C_max_, ng/ml), time to peak milk concentration (T_max_, h), curve of area under the concentration vs. time extrapolated to infinity (AUC_last_, μg-h/ml), elimination half-life (T_½_ el, h), minimum inhibitory concentration (MIC, μg/ml), relationship between the maximum concentration and MIC (C_max_/MIC), relation between the area under the curve and MIC (AUC/MIC), amount of time that concentration level is above MIC (T > MIC, h)*.

*The treatment groups correspond with the following formulations: A1 (600 mg/ml of Aloe vera + 0.25 mg/ml of cloxacillin), A2 (600 mg/ml of Aloe vera + 0.5 mg/ml of cloxacillin), A3 (600 mg/ml of Aloe vera + 0.25 mg/ml of ceftiofur), and A4 (600 mg/ml of Aloe vera + 0.5 mg/ml of ceftiofur)*.

*Efficacy indices of cloxacillin and ceftiofur were calculated based on the minimum inhibitory concentration (MIC) against S. aureus ATCC 29213 as calculated for the combination Aloe vera + antimicrobial (0.06 μg/ml for cloxacillin and ceftiofur)*.

### Minimum Inhibitory Concentration Testing

When tested against *S. aureus*, the formulations combining extract and cloxacillin or ceftiofur showed MIC values of 20 mg/ml for *Aloe vera* and 0.06 μg/ml for cloxacillin and ceftiofur. Contrarily, the MIC when these compounds were not combined was 40 mg/ml for the extract and 0.25 μg/ml for cloxacillin and ceftiofur.

### Efficacy Indices

Tables 4, 5 show the values observed for the efficacy indices of each formulation. As seen in Table 4, three therapeutic efficacy indices were calculated for aloin in all the treatment groups. However, considering that T > MIC is the efficacy index recommended for β-lactams (see Table 5), this index was the only one calculated for cloxacillin in the treatment groups A1 and A2, and for ceftiofur in the treatment groups A3 and A4.

These efficacy indices were calculated based on both the PK parameters for each formulation and the MIC values for each antimicrobial. In the case of aloin, its MIC value (3.66 ng/ml) was calculated based on the concentration of aloin in the *Aloe vera* extract.

### Safety Assessment

As in the PK analysis, samples for safety assessment were obtained from eight quarters for each formulation, except for the treatment group A4 due to one dysfunctional teat.

The number of quarters presenting milk abnormalities after administering the intramammary treatments increased progressively in four treatment groups. The percentage of affected quarters was 65, 65, 90, and 73.8% for A1, A2, A3, and A4, respectively. Nevertheless, cows made a full recovery as the number of reactive quarters decreased after 96 h. The safety assessment for all four formulations (combining *Aloe vera* and cloxacillin or ceftiofur) also included measuring the SCC. In that regard, Table 6 shows the transformation of SCC into SCS for the sampling points at 24, 48, 72, and 96 h. As seen in the table, SCS increased in all quarters after administering treatments, though milk somatic cells decreased progressively after 24 h. The effect of time was significant for every treatment (*p* = 0.003), with the exception of the difference between the SCS values at 48 and 72 h (*p* = 0.334). In addition, the treatment group A3 showed lower SCS values than the other groups, but the differences between groups were not significant (*p* = 0.069).

**Table 6:** Mean ± SD of the quarter mean somatic cell score (QSCS) (*n* = 8 per groups A1, A2, and A3 and *n* = 7 per group A4), before and after administering treatments. Somatic cell count (SCC) of each quarter selected for safety assessment of intramammary formulations, logarithmically transformed into SCS before and every 24 h after a single dose.

	**QSCS pre-treatment**	**QSCS 24 h**	**QSCS 48 h**	**QSCS 72 h**	**QSCS 96 h**
A1	10.89 ± 0.81	16.85 ± 1.62	15.57 ± 1.51	15.25 ± 1.48	13.131 ±.92
A2	10.10 ± 0.34	16.95 ± 1.81	15.44 ± 2.11	15.20 ± 1.60	13.66 ± 1.17
A3	9.20 ± 0.31	14.75 ± 0.76	13.38 ± 1.28	14.00 ± 0.94	11.62 ± 0.53
A4	11.00 ± 1.57	16.18 ± 2.75	15.70 ± 3.67	14.87 ± 2.75	13.42 ± 2.34

*Treatment groups correspond with the following formulations: A1 (600 mg/ml of Aloe vera + 0.25 mg/ml of cloxacillin), A2 (600 mg/ml of Aloe vera + 0.5 mg/ml of cloxacillin), A3 (600 mg/ml of Aloe vera + 0.25 mg/ml of ceftiofur), and A4 (600 mg/ml of Aloe vera + 0.5 mg/ml of ceftiofur)*.

All four treatments tested positive for bacterial growth in some quarters, at least on one sampling point. However, different pathogens were identified in those cases that tested positive in two consecutive cultures from the same quarter. Therefore, bacteriologic cultures were not conclusive in terms of suggesting that an intramammary infection was linked to the administration of any treatment.

## Discussion

In this study, two formulations with the *Aloe vera* extract combined with low doses of cloxacillin and ceftiofur reached concentrations in milk that indicate inhibitory efficacy against *S. aureus*. When combined, this considers a significant reduction of MIC values for cloxacillin or ceftiofur and the *Aloe vera* extract against *S. aureus, in vitro*, when compared with the MIC values determined separately. Therefore, the combinations between *Aloe vera* and cloxacillin or ceftiofur at determined concentrations might be an effective therapy to treat bovine mastitis caused by *S. aureus* after a single dose.

The possibility that active ingredients in medicinal compounds could potentiate their therapeutic activities—thus requiring lower doses of each active ingredient when combined than what it would be recommended on their own—has been noted by the European Medicines Agency (EMA). Hence, the Agency provided a guide for the assessment of fixed combination of herbal medicine products (49) that specifically encourages considering PD interactions between compounds and the addition of adverse effects.

The success of any antibacterial therapy is influenced by PK characteristics of an antimicrobial, and in the case of intramammary formulations commonly used for the treatment of bovine mastitis, their action mechanism is mostly time dependent. This means that drug concentrations at the infection site must remain above the MIC for as long as possible to ensure maximum efficacy (31, 38, 50). Consequently, time above MIC (T > MIC) is the appropriate efficacy index for the purpose of predicting the therapeutic effect of this kind of antimicrobials [e.g., β-lactams, such as cloxacillin and ceftiofur (33, 51–53)]. Noticeably, even though no efficacy index has been described for aloin, a time-dependent action has been reported for *Aloe vera* (54).

As for the remaining indices of the PK/PD analysis, these were calculated for all four formulations using the PK/PD parameters of anthraquinones (aloins A and B) in milk, as they were deemed the main antimicrobial drugs in the *Aloe vera* gel extract. These formulations showed differences in their PK parameters that might relate to the concentration of antimicrobial drugs used in each formula. In the case of treatment groups A2 and A4, the *C*_max_ value for cloxacillin and ceftiofur were 4.17 and 1.02 μg/ml of milk, respectively, whereas for groups A1 and A3 their *C*_max_ values were of 0.84 and 0.94 μg/ml of milk, respectively. Interestingly, even when all formulations included aloin (A + B) at the same concentration (109.7 ng/ml), those groups that received formulations containing 0.25 mg/ml of cloxacillin or ceftiofur (A1 and A3) showed higher concentrations of aloin in milk than those that received formulations containing 0.5 mg/ml of cloxacillin or ceftiofur (groups A2 and A4). Specifically, *C*_max_ for groups A1, A3, A2, and A4 were 37.84, 90.17, 15.08, and 59.08 ng/ml, respectively, and these values peaked close to 3 h after administering each treatment.

A possible explanation for such variability might be the precipitation of *Aloe vera* compounds when combined with higher concentrations of antibiotics, especially cloxacillin, due to some incompatibility with this antimicrobial (55). Such precipitation could imply lower distribution of the active compounds, as well as prolonged exposure of the mammary gland tissues to the irritating effect of these drugs, and resulting in lesser drug recovery through milking (56). The absence of *Aloe vera* and antibiotic controls might be considered as a limitation of this first study because such controls could be useful to elucidate further variability. Nevertheless, these controls were excluded due to neither of these antibacterial agents milk concentrations could reach recommended values for the efficacy indices after a single dose, at the same concentration of the experimental formulations, as compared when the MIC values of compound used individually are considered. Therefore, further conclusive interpretations in this regard would require administering control formulations including *Aloe vera*, cloxacillin, and ceftiofur separately, followed by an assessment of their PK parameters.

Notwithstanding, our data show that T > MIC values for groups A1 and A3 met the recommended level for aloin, cloxacillin, or ceftiofur (14, 12, and 17 h, respectively) when a dosing interval of 24 h was considered. Aloin T > MIC values were 23.29 and 27.5 h, whereas cloxacillin and ceftiofur were 19.20 and 19.74 h for the A1 and A3 groups, respectively. In addition, some studies have reported that anthraquinones and tetracycline of *Aloe vera* show a similar mechanism of action, inhibiting protein synthesis by blocking binding to the bacterial ribosome A, which results in nil bacterial growth in culture media containing the *Aloe vera* extract (57). Doxycycline is one example from the tetracyclines class that has been reported with such similarity (58); thus, an alternative reference value for predicting the therapeutic efficacy of aloin could be an AUC/MIC > 25. Under that criterion, all formulations reached the optimum value for this recommended value.

It is important to remark that MIC values against *S. aureus* ATCC 29213 (a methicillin-sensitive strain) were considered a standard value in this preliminary study because no differences in susceptibility to *Aloe vera* + cloxacillin combination were presented by methicillin-resistant reference strain, *S. aureus* ATCC 43300. Nevertheless, further efficacy studies of improved formulations based on these results should include MIC values obtained from on-farm intramammary strains (59, 60).

Some adverse effects have also been reported for oral or topic administration of *Aloe vera*, including some hypersensitivity reactions (61) and are most commonly linked to specific compounds like its anthraquinones. For instance, products derived from *Aloe vera* should not contain more than 10 μg/g of aloin when they are administered orally, due to its potent laxative effect. In contrast, the limit for products administered topically or by other routes can be raised up to 50 μg/g of aloin. However, toxicity studies for *Aloe vera* found no difference between whole-leave and purified aloin products containing the same anthraquinones concentration used in our formulations (>0.1 μg/g). Bearing this information in mind, maybe anthraquinones are rather markers for another compound instead of a toxicity source themselves (62).

Meanwhile, some reports have described that intramammary formulations of *Aloe vera* and other plants show an anti-inflammatory effect (25), but it might relate to specific farming conditions of dairy cattle. One of these formulations is currently available as a commercial product (Mastiliber^®^), though it is only registered for use in Mexico. After using this product in our research, all treatment groups showed an altered milk appearance and elevated SCS, possibly due to hypersensitivity reactions linked to *Aloe vera*. This, in spite of the aloin concentration of 109.71 ng/ml in the intramammary formulations, is lower than the recommended dose limit. Therefore, for the use of a standardized *Aloe vera* product, it is important to warrantee that the concentration of aloin in the intramammary formulations are safe and effective.

It is worth noting that a common indicator to assess milk quality, hygiene, udder health, and dairy cow welfare is the SCC, which allows detecting an infected udder quarter when its value reaches the threshold of 200,000 cells/ml, even before the affected quarter shows clinical signs of infection (63). These somatic cells include both those shed from the secretory tissue as well as leukocytes, and though the latter are mostly fighting infections, they also help with the repairing of damaged tissues, increasing their numbers along with the inflammatory response of the udder (64–66). For instance, some studies have reported that using some intramammary formulations (based on different active principles) in cows and goats results in a marked increase in SCS that subsides progressively after 12 h, whether the animals are coursing or not with clinical mastitis (56, 67).

In our study, the animals that showed the most severe signs of irritation at quarter level were those in the two groups that received formulations containing cloxacillin. This finding could be linked to a lower solubility of this drug and its incompatibility with several compounds, which in turn reduces the solubility of combined formulas (55). Hence, the two formulations containing cloxacillin could results in lower drug distribution and prolonged contact with mammary tissues, leading to acute inflammation and a gradual recovery afterward. However, to better assess the impact of both the active principles and the mechanic effect triggered by the administration with intramammary syringes, further studies must consider administering only a vehicle solution to a control group. In addition, clinical and hematological testing must be performed at the whole cow level for the safety assessment (56, 68).

In conclusion, the alternative treatment regimens for mastitis or intramammary infections in dairy cows caused by *S. aureus* could also consider certified combinations of *Aloe vera* and low doses of β-lactams, such as cloxacillin and ceftiofur. These β-lactams proposed doses are lower than those currently found in commercially available intramammary products and informed by the values of the PK/PD parameters commonly used to predict the therapeutic efficacy of the most frequently used intramammary drugs. Therefore, the combinations of *Aloe vera* and low doses of cloxacillin or ceftiofur could also mean a potential reduction of the withdrawal period and a lower risk of antibiotic residue violations in bulk milk tank, thus contributing to the accomplishment of safety food standards, without reducing efficacy. In addition, the several action mechanisms of the *Aloe vera* extract against bacteria, and a synergistic interaction between its combined compounds, could delay bacterial resistance in comparison with the sole use of antibiotics. However, extreme caution must be taken in the use of domestic on-farm *Aloe vera* preparations to avoid mammary tissue damage or treatment failures, as a consequence of using a non-standardized preparation and not a safety and efficacy tested product. The present results, consequently, were obtained for the particular formulations described, and other formulations should be validated and pharmacologically explored as well. Additionally, it is important to note that the current study has been focused on the efficacy of these combinations against *S. aureus*, and the clinical efficacy of the *Aloe vera*-antibiotic combinations against bacteria different than *S. aureus* should be individually assessed.

Following the finding of this study, further studies for the improvement of these formulations to guarantee their safety and stability, as well as the assessment of *in vivo* efficacy using *S. aureus* intramammary infection isolated strain and optimal clinical therapeutic schedule to complete PK studies are warranted.

## Data Availability Statement

The original contributions presented in the study are included in the article/supplementary material, further inquiries can be directed to the corresponding author/s.

## Ethics Statement

The animal study was reviewed and approved by Program of the Animal Care and Use Institutional Committee (CICUA) of Universidad de Chile.

## Author Contributions

NF-B ran the project, including the preparation of intramammary formulations, administration of treatments, collection of samples, safety assessment, analytical procedures, data analyses, evaluation and interpretation of the results, and contributed to drafting the manuscript. OC contributed to experimental assays. MM administered treatments, collected samples, performed safety assessments, and made important contributions to the revision of the draft manuscript. PP administered treatments, collected samples, and performed safety assessments. DI ran the project, including drafting the experimental design, evaluation, and interpretation of results. JC contributed to the revision of the draft manuscript. BS supervisor and project manager, was responsible for designing the study, as well as the analytical procedure, and contributed to the revision of the draft manuscript. All authors state that they have read and approved the final manuscript.

## Conflict of Interest

The authors declare that the research was conducted in the absence of any commercial or financial relationships that could be construed as a potential conflict of interest.
